# Crystal structure and Hirshfeld surface analysis of (*E*)-2-(4-bromo­phen­yl)-1-[2,2-di­bromo-1-(4-nitro­phen­yl)ethen­yl]diazene

**DOI:** 10.1107/S205698902200620X

**Published:** 2022-06-16

**Authors:** Mehmet Akkurt, Sema Öztürk Yıldırım, Namiq Q. Shikhaliyev, Naila A. Mammadova, Ayten A. Niyazova, Victor N. Khrustalev, Ajaya Bhattarai

**Affiliations:** a Department of Physics, Faculty of Science, Erciyes University, 38039 Kayseri, Turkey; b Department of Physics, Faculty of Science, Eskisehir Technical University, Yunus Emre Campus 26470 Eskisehir, Turkey; c Organic Chemistry Department, Baku State University, Z. Khalilov str. 23, AZ 1148 Baku, Azerbaijan; d Azerbaijan State University of Economics (UNEC), Istiglaliyyat str., Baku, Azerbaijan; e Peoples’ Friendship University of Russia (RUDN University), Miklukho-Maklay St. 6, Moscow, 117198, Russian Federation; f N. D. Zelinsky Institute of Organic Chemistry RAS, Leninsky Prosp. 47, Moscow, 119991, Russian Federation; g Department of Chemistry, M.M.A.M.C (Tribhuvan University) Biratnagar, Nepal

**Keywords:** crystal structure, non-covalent inter­actions, C—H⋯Br inter­actions, *C*(8) chains, Hirshfeld surface analysis

## Abstract

The mol­ecule of the title compound, C_14_H_8_Br_3_N_3_O_2_, consists of three almost planar groups: the central di­bromo­ethenyldiazene fragment and two attached aromatic rings. The mean planes of these rings form dihedral angles with the plane of the central fragment of 26.35 (15) and 72.57 (14)° for bromine- and nitro-substituted rings, respectively. In the crystal, C—H⋯Br inter­actions connect mol­ecules, generating zigzag *C*(8) chains along the [100] direction. These chains are linked by C—Br⋯π inter­actions into layers parallel to (001). van der Waals inter­actions between the layers aid in the cohesion of the crystal packing. The most substantial contributions to crystal packing, according to a Hirshfeld surface analysis, are from Br⋯H/H⋯Br (20.9%), C⋯H/H⋯C (15.2%), O⋯H/H⋯O (12.6%) and H⋯H (11.7%) contacts.

## Abstract 2

C—H⋯Br inter­actions connect mol­ecules in the crystal, resulting in zigzag *C*(8) chains along the [100] direction. C—Br⋯π inter­actions connect these chains into parallel layers to (002). van der Waals inter­actions between the layers aid in the cohesion of the crystal packing.

## 1. Chemical context

Azo dyes constitute the largest production volume (*ca* 70%) of the dye industry today, and their relative importance may increase further in the future (Lipskikh *et al.*, 2018 ▸). They play a crucial role in the printing market, the design of functional materials attributed to smart hydrogen bonding, photo-triggered structural switching, self-assembled layers, ionophores, liquid crystals, semiconductors, indicators, spectrophotometric reagents for determination of metal ions, photoluminescent materials, catalysts, anti­microbial agents, optical recording media, spin-coating films, *etc* (Zollinger, 1994 ▸, 1995 ▸; Gurbanov *et al.*, 2020*a* ▸,*b* ▸; Mahmudov *et al.*, 2010 ▸, 2013 ▸). Depending on the attached substituents, the functional properties of azo compounds and their metal complexes can be improved/controlled (Ma *et al.*, 2020 ▸, 2021 ▸). Both *E*/*Z* isomerism and azo–hydrazo tautomerism properties of azo dyes are key phenomena in the synthesis and development of new functional materials (Shixaliyev *et al.*, 2018 ▸, 2019 ▸). The attachment of non-covalent bond acceptor or donor centres to the azo dyes can be used as a synthetic strategy for the improvement of the functional properties of their metal complexes (Mahmudov *et al.*, 2020 ▸, 2021 ▸, 2022 ▸). Thus, we have attached bromine atoms and a nitro group together with aryl rings to the –N=N– linkage leading to a new azo compound, (*E*)-2-(4-bromo­phen­yl)-1-[2,2-di­bromo-1-(4-nitro­phen­yl)ethen­yl]diazene, which can provide inter­molecular halogen and hydrogen bonds as well as π-inter­actions. 
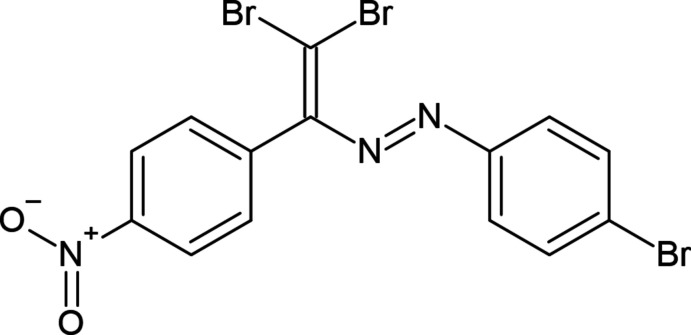


## 2. Structural commentary

**Figure 1:**
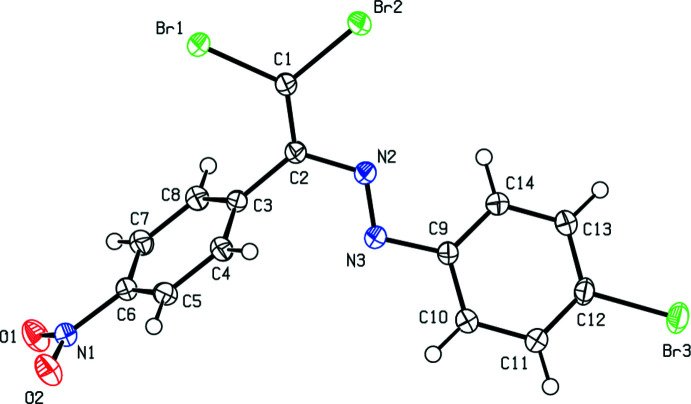
The mol­ecular structure of the title compound. Displacement ellipsoids are drawn at the 50% probability level.

The mol­ecule of the title compound (Fig. 1 ▸) consists of three almost planar groups: the central di­bromo­ethenyldiazene fragment [largest deviation from the l.s. plane is 0.039 (3) Å for N2] and two attached aromatic rings. The mean planes of these rings form dihedral angles with the plane of the central fragment of 26.35 (15) and 72.57 (14)° for the bromine- and nitro-substituted rings, respectively. The nitro group is twisted by 8.1 (2)° with respect to the C3–C8 aromatic ring. The C2—N2 bond distance of 1.406 (4) Å indicates π-conjugation between ethene and diazo groups. All other bond lengths and angles in the title compound are similar to those reported for the related azo compounds discussed in the *Database survey* section.

## 3. Supra­molecular features and Hirshfeld surface analysis

**Figure 2:**
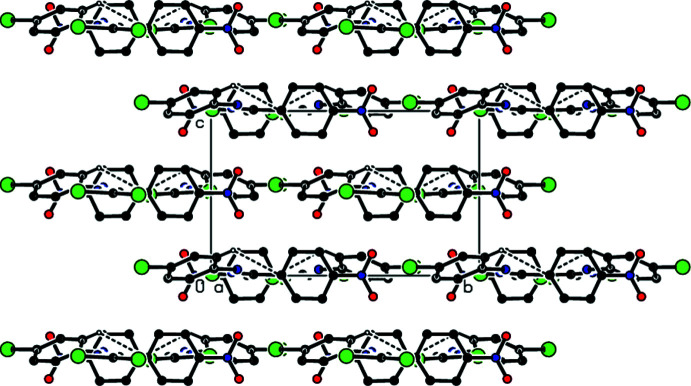
View down the *a*-axis of the title compound showing the C—H⋯Br inter­actions.

**Figure 4:**
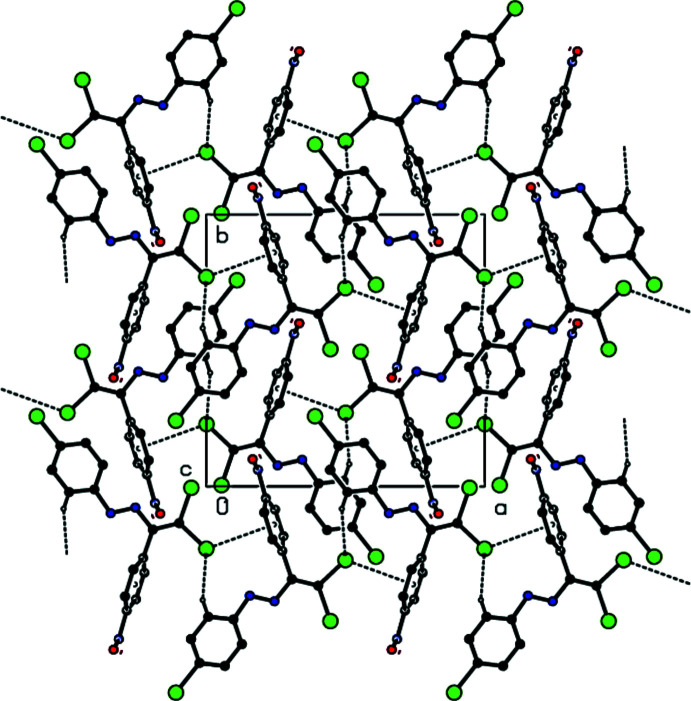
View down the *c* axis of the title compound, showing the C—Br⋯π inter­actions.

In the crystal, C—H⋯Br inter­actions connect the mol­ecules, generating zigzag *C*(8) chains (Bernstein *et al.*, 1995 ▸) along the [100] direction (Table 1 ▸, Figs. 2 ▸ and 3 ▸). These chains are linked by C—Br⋯π inter­actions [C1—Br1⋯*Cg*1^ii^; C1—Br1 = 1.864 (3) Å, Br1⋯*Cg*1^ii^ = 3.5803 (16) Å, C1⋯*Cg*1^ii^ = 4.722 (3) Å, C1—Br1⋯*Cg*1^ii^ = 116.77 (9)°; *Cg*1 is the centroid of the C3–C8 ring; symmetry code (ii): *x* + 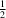 , −*y* + 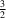 , *z*] into layers parallel to (001) (Fig. 4 ▸). van der Waals inter­actions between the layers help to keep the crystal packing together.

**Figure 5:**
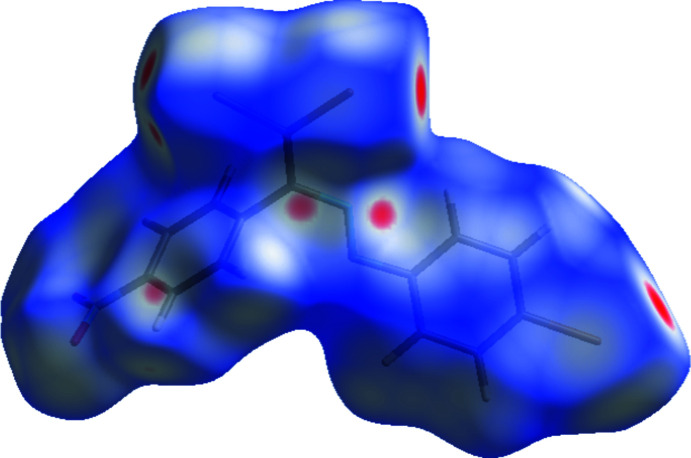
View of the three-dimensional Hirshfeld surface of the title compound plotted over *d*_norm_ in the range −0.1401 to 1.1158 a.u.

*Crystal Explorer 17.5* (Turner *et al.*, 2017 ▸) was used to perform a Hirshfeld surface analysis and to generate the corresponding two-dimensional fingerprint plots, with a standard resolution of the three-dimensional *d*_norm_ surfaces plotted over a fixed color scale of −0.1401 (red) to 1.1158 (blue) a.u. (Fig. 5 ▸). The red patches represent short contacts and negative *d*_norm_ values on the surface, which correspond to the C—H⋯Br hydrogen bonds discussed above (Table 1 ▸). The C10—H10⋯Br1 inter­actions, which are important for mol­ecular packing of the title compound, are responsible for the red patch that appears around Br1.

**Figure 6:**
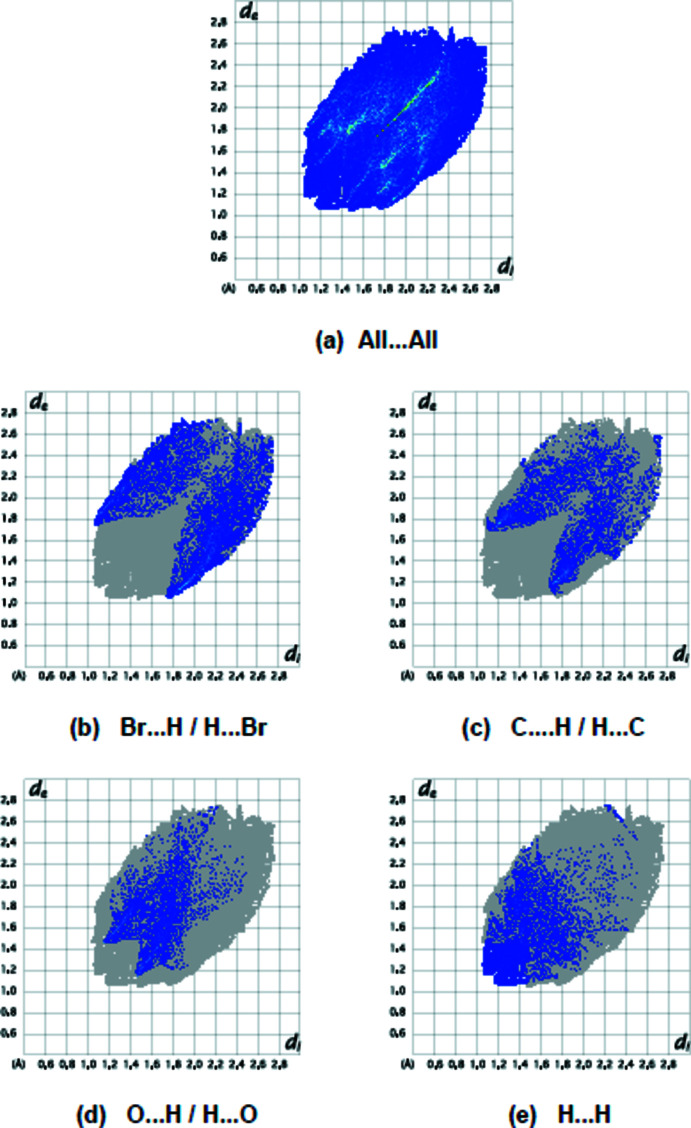
The full two-dimensional fingerprint plots for the title compound, showing all inter­actions (*a*) and delineated into (*b*) Br⋯H/H⋯Br, (*c*) C⋯H/H⋯C, (*d*) O⋯H/H⋯O, and (*e*) H⋯H inter­actions. The *d*_i_ and *d*_e_ values are the closest inter­nal and external distances (in Å) from given points on the Hirshfeld surface.

The overall two-dimensional fingerprint plot for the title compound and those delineated into Br⋯H / H⋯Br (20.9%), C⋯H/H⋯C (15.2%), O⋯H/H⋯O (12.6%) and H⋯H (11.7%) contacts are shown in Fig. 6 ▸, while numerical details for short inter­molecular contacts are given in Table 2 ▸. Br⋯C/C⋯Br (8.8%), Br⋯Br (6.7%), N⋯H/H⋯N (6.5%), Br⋯O/O⋯Br (5.6%), O⋯C/C⋯O (4.1%), Br⋯N/N⋯Br (3.9%), C⋯C (2.5%), O⋯N/N⋯O (1.3%) and N⋯C/C⋯N (0.1%) contacts have little directional influence on the mol­ecular packing.

## 4. Database survey

A search of the Cambridge Structural Database (CSD, Version 5.42, update of September 2021; Groom *et al.*, 2016 ▸) for similar structures with the (*E*)-1-(2,2-di­bromo-1-phenyl­ethen­yl)-2-phenyl­diazene fragment showed that the nine closest are those of CSD refcodes TAZDIL [(**I**); Atioğlu *et al.*, 2022 ▸], PAXDOL [(**II**); Çelikesir *et al.*, 2022 ▸], GUPHIL [(**III**); Özkaraca *et al.*, 2020*b* ▸], HONBUK [(**IV**); Akkurt *et al.*, 2019 ▸], HONBOE [(**V**); Akkurt *et al.*, 2019 ▸], HODQAV [(**VI**); Shikhaliyev *et al.*, 2019 ▸], XIZREG [(**VII**); Atioğlu *et al.*, 2019 ▸], LEQXOX [(**VIII**); Shikhaliyev *et al.*, 2018 ▸] and LEQXIR [(**IX**); Shikhaliyev *et al.*, 2018 ▸].

In (**I**), the mol­ecules are connected by C—H⋯O and C—H⋯F hydrogen bonds into layers parallel to (011). The crystal packing is consolidated by C—Br⋯π and C—F⋯π contacts, as well as by π–π stacking inter­actions. In the crystal of (**II**), the mol­ecules are linked into chains running parallel to [001] by C—H⋯O hydrogen bonds. The crystal packing is consolidated by C—F⋯π contacts and π–π stacking inter­actions, and short Br⋯O [2.9828 (13) Å] distances are also observed. In the crystal of (**III**), the mol­ecules are linked into inversion dimers *via* short halogen–halogen contacts [Cl1⋯Cl1 = 3.3763 (9) Å, C16—Cl1⋯Cl1 = 141.47 (7)°] compared to the van der Waals radius sum of 3.50 Å for two chlorine atoms. No other directional contacts could be identified, and the shortest aromatic ring centroid separation is greater than 5.25 Å. In the crystals of (**IV**) and (**V**), the mol­ecules are linked through weak *X*⋯Cl contacts [*X* = Cl for (**IV**) and Br for (**V**)], C—H⋯Cl and C—Cl⋯π inter­actions into sheets lying parallel to (001). In the crystal of (**VI**), the mol­ecules are stacked in columns parallel to [100] *via* weak C—H⋯Cl hydrogen bonds and face-to-face π–π stacking inter­actions. The crystal packing is further consolidated by short Cl⋯Cl contacts. In (**VII**), mol­ecules are linked by C—H⋯O hydrogen bonds into zigzag chains running parallel to [001]. The crystal packing also features C—Cl⋯π, C—F⋯π and N—O⋯π inter­actions. In (**VIII**), C—H⋯N and short Cl⋯Cl contacts are observed, and in (**IX**), C—H⋯N and C—H⋯O hydrogen bonds and short Cl⋯O contacts occur.

## 5. Synthesis and crystallization

This dye was synthesized according to the reported method (Akkurt *et al.*, 2019 ▸; Atioğlu *et al.*, 2019 ▸; Maharramov *et al.*, 2018 ▸; Özkaraca *et al.*, 2020*a* ▸,*b* ▸). A 20 mL screw neck vial was charged with DMSO (10 mL), (*E*)-1-(4-bromo­phen­yl)-2-(4-nitro­benzyl­idene)hydrazine (1 mmol), tetra­methyl­ethylene­di­amine (TMEDA; 295 mg, 2.5 mmol), CuCl (2 mg, 0.02 mmol) and CBr_4_ (4.5 mmol). After 1-3 h (until TLC analysis showed complete consumption of corresponding Schiff base), the reaction mixture was poured into 0.01 *M* solution of HCl (100 mL, pH = 2–3), and extracted with di­chloro­methane (3 × 20 mL). The combined organic phase was washed with water (3 × 50 mL), brine (30 mL), dried over anhydrous Na_2_SO_4_ and concentrated *in vacuo* using a rotary evaporator. The residue was purified by column chromatography on silica gel using appropriate mixtures of hexane and di­chloro­methane (3/1–1/1). Crystals suitable for X-ray analysis were obtained by slow evaporation of an ethanol solution. Red solid (58%); m.p. 398 K. Analysis calculated for C_14_H_8_Br_3_N_3_O_2_ (*M* = 489.95): C 34.32, H 1.65, N 8.58; found: C 34.27, H 1.70, N 8.56%. ^1^H NMR (300 MHz, CDCl_3_) *δ* 8.16–7.41 (8H, Ar–H). ^13^C NMR (75MHz, CDCl_3_) *δ* 150.89, 149.62, 148.26, 136.43, 132.25, 127.77, 125.57, 124.53, 123.57, 93.24. ESI–MS: *m*/*z*: 490.96 [*M* + H]^+^.

## 6. Refinement

Crystal data, data collection and structure refinement details are summarized in Table 3 ▸. All H atoms were positioned geometrically and constrained to ride on their parent atoms (C—H = 0.95 Å) with *U*_iso_(H) = 1.2*U*_eq_(C). One reflection (110), affected by the beam stop, was omitted in the final cycles of refinement.

## Supplementary Material

Crystal structure: contains datablock(s) I. DOI: 10.1107/S205698902200620X/yk2170sup1.cif

Structure factors: contains datablock(s) I. DOI: 10.1107/S205698902200620X/yk2170Isup2.hkl

Supporting information file. DOI: 10.1107/S205698902200620X/yk2170Isup3.cml

CCDC reference: 2178832

Additional supporting information:  crystallographic information; 3D view; checkCIF report

## Figures and Tables

**Figure 3:**
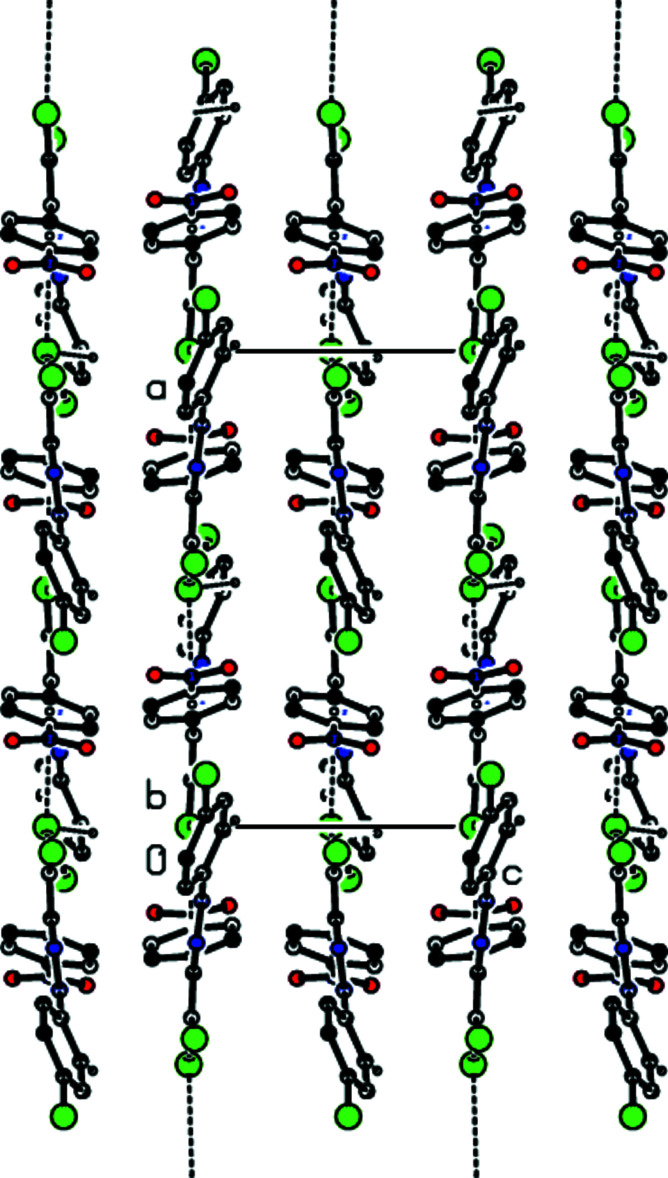
View down the *b*-axis of the title compound, showing the C—Br⋯π inter­actions.

**Table 1:** Hydrogen-bond geometry (Å, °)

*D*—H⋯*A*	*D*—H	H⋯*A*	*D*⋯*A*	*D*—H⋯*A*
C10—H10⋯Br1^i^	0.95	2.89	3.530 (4)	126

Symmetry code: (i) 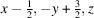 .

**Table 2:** Summary of short inter­atomic contacts (Å) in the title compound

Br1⋯H10	2.89	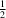 + *x*, 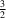 − *y*, *z*
H14⋯H5	2.40	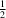 − *x*, − 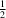 + *y*, − 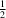 + *z*
Br2⋯Br3	3.44	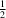 + *x*, 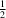 − *y*, *z*
H10⋯C13	3.02	−*x*, 1 − *y*, 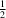 + *z*
O1⋯H13	2.75	*x*, 1 + *y*, *z*
H7⋯N2	2.65	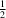 − *x*, 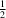 + *y*, − 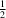 + *z*

**Table 3:** Experimental details

Crystal data	
Chemical formula	C_14_H_8_Br_3_N_3_O_2_
*M*_r_	489.96
Crystal system, space group	Orthorhombic, *P* *n* *a*2_1_
Temperature (K)	100
*a*, *b*, *c* (Å)	13.8678 (5), 13.5442 (5), 8.3017 (3)
*V* (Å^3^)	1559.29 (10)
*Z*	4
Radiation type	Mo *K*α
μ (mm^−1^)	7.77
Crystal size (mm)	0.31 × 0.14 × 0.08

Data collection	
Diffractometer	Bruker D8 QUEST, Photon III detector
Absorption correction	Multi-scan (*SADABS*; Krause *et al.*, 2015 ▸)
*T*_min_, *T*_max_	0.044, 0.110
No. of measured, independent and observed [*I* > 2σ(*I*)] reflections	75835, 7370, 5962
*R*_int_	0.057
(sin θ/λ)_max_ (Å^−1^)	0.826

Refinement	
*R*[*F* ^2^ > 2σ(*F* ^2^)], *wR*(*F* ^2^), *S*	0.033, 0.085, 1.02
No. of reflections	7370
No. of parameters	199
No. of restraints	1
H-atom treatment	H-atom parameters constrained
Δρ_max_, Δρ_min_ (e Å^−3^)	1.41, −0.97
Absolute structure	Flack parameter determined using 2437 quotients [(*I* ^+^)−(*I* ^−^)]/[(*I* ^+^)+(*I* ^−^)] (Parsons *et al.*, 2013 ▸).
Absolute structure parameter	0.003 (5)

Computer programs: *APEX3* and *SAINT* (Bruker, 2018 ▸), *SHELXT* (Sheldrick, 2015*a* ▸), *SHELXL2018* (Sheldrick, 2015*b* ▸), *ORTEP-3 for Windows* (Farrugia, 2012 ▸) and *PLATON* (Spek, 2020 ▸).

## supplementary crystallographic information

### Crystal data

**Table:**

C_14_H_8_Br_3_N_3_O_2_	*D*_x_ = 2.087 Mg m^−^^3^
*M**_r_* = 489.96	Mo *K*α radiation, λ = 0.71073 Å
Orthorhombic, *P**n**a*2_1_	Cell parameters from 9976 reflections
*a* = 13.8678 (5) Å	θ = 2.9–34.8°
*b* = 13.5442 (5) Å	µ = 7.77 mm^−^^1^
*c* = 8.3017 (3) Å	*T* = 100 K
*V* = 1559.29 (10) Å^3^	Block, red
*Z* = 4	0.31 × 0.14 × 0.08 mm
*F*(000) = 936	

### Data collection

**Table:**

Bruker D8 QUEST, Photon III detector diffractometer	7370 independent reflections
Radiation source: fine-focus sealed X-Ray tube	5962 reflections with *I* > 2σ(*I*)
Graphite monochromator	*R*_int_ = 0.057
Detector resolution: 7.31 pixels mm^-1^	θ_max_ = 36.0°, θ_min_ = 2.9°
φ and ω shutterless scans	*h* = −22→22
Absorption correction: multi-scan (SADABS; Krause *et al.*, 2015)	*k* = −22→22
*T*_min_ = 0.044, *T*_max_ = 0.110	*l* = −13→13
75835 measured reflections	

### Refinement

**Table:**

Refinement on *F*^2^	Secondary atom site location: difference Fourier map
Least-squares matrix: full	Hydrogen site location: inferred from neighbouring sites
*R*[*F*^2^ > 2σ(*F*^2^)] = 0.033	H-atom parameters constrained
*wR*(*F*^2^) = 0.085	*w* = 1/[σ^2^(*F*_o_^2^) + (0.0438*P*)^2^ + 0.8309*P*] where *P* = (*F*_o_^2^ + 2*F*_c_^2^)/3
*S* = 1.02	(Δ/σ)_max_ = 0.002
7370 reflections	Δρ_max_ = 1.41 e Å^−^^3^
199 parameters	Δρ_min_ = −0.97 e Å^−^^3^
1 restraint	Absolute structure: Flack parameter determined using 2437 quotients [(*I*^+^)-(*I*^-^)]/[(*I*^+^)+(*I*^-^)] (Parsons *et al.*, 2013).
Primary atom site location: structure-invariant direct methods	Absolute structure parameter: 0.003 (5)

### Special details

**Table:**

Geometry. All esds (except the esd in the dihedral angle between two l.s. planes) are estimated using the full covariance matrix. The cell esds are taken into account individually in the estimation of esds in distances, angles and torsion angles; correlations between esds in cell parameters are only used when they are defined by crystal symmetry. An approximate (isotropic) treatment of cell esds is used for estimating esds involving l.s. planes.
Refinement. Refinement of F^2^ against ALL reflections. The weighted R-factor wR and goodness of fit S are based on F^2^, conventional R-factors R are based on F, with F set to zero for negative F^2^. The threshold expression of F^2^ > 2sigma(F^2^) is used only for calculating R-factors(gt) etc. and is not relevant to the choice of reflections for refinement. R-factors based on F^2^ are statistically about twice as large as those based on F, and R- factors based on ALL data will be even larger.

### Fractional atomic coordinates and isotropic or equivalent isotropic displacement parameters (Å^2^)

**Table:**

	*x*	*y*	*z*	*U*_iso_*/*U*_eq_	
Br1	0.49964 (2)	0.72932 (3)	0.49158 (6)	0.02765 (8)	
Br2	0.44572 (2)	0.50470 (2)	0.51189 (7)	0.02906 (8)	
Br3	−0.10955 (3)	0.24165 (3)	0.55241 (7)	0.03182 (9)	
O1	0.1810 (2)	1.1045 (2)	0.3729 (4)	0.0333 (6)	
O2	0.1662 (3)	1.1002 (2)	0.6323 (4)	0.0326 (6)	
N1	0.18506 (19)	1.0613 (2)	0.5020 (5)	0.0231 (5)	
N2	0.24431 (19)	0.57755 (19)	0.5188 (4)	0.0207 (5)	
N3	0.15599 (19)	0.5982 (2)	0.5376 (4)	0.0228 (6)	
C1	0.4020 (2)	0.6350 (2)	0.5012 (6)	0.0222 (6)	
C2	0.3073 (2)	0.6586 (2)	0.5080 (5)	0.0205 (5)	
C3	0.2727 (2)	0.7629 (2)	0.5040 (5)	0.0196 (5)	
C4	0.2373 (3)	0.8055 (3)	0.6449 (5)	0.0221 (6)	
H4	0.233234	0.767615	0.741062	0.027*	
C5	0.2076 (3)	0.9043 (3)	0.6441 (5)	0.0222 (6)	
H5	0.184039	0.934772	0.739348	0.027*	
C6	0.2136 (2)	0.9563 (2)	0.5016 (5)	0.0212 (5)	
C7	0.2468 (3)	0.9148 (3)	0.3596 (5)	0.0249 (7)	
H7	0.248972	0.952162	0.262834	0.030*	
C8	0.2769 (3)	0.8171 (3)	0.3629 (5)	0.0235 (6)	
H8	0.300598	0.787169	0.267351	0.028*	
C9	0.0971 (2)	0.5110 (2)	0.5422 (5)	0.0208 (6)	
C10	0.0066 (3)	0.5207 (3)	0.6113 (5)	0.0251 (7)	
H10	−0.013356	0.582330	0.654399	0.030*	
C11	−0.0553 (3)	0.4391 (3)	0.6172 (5)	0.0262 (7)	
H11	−0.117165	0.444374	0.665505	0.031*	
C12	−0.0249 (3)	0.3509 (2)	0.5516 (5)	0.0243 (6)	
C13	0.0662 (2)	0.3397 (2)	0.4837 (5)	0.0246 (7)	
H13	0.085933	0.277960	0.440465	0.030*	
C14	0.1275 (2)	0.4203 (2)	0.4803 (5)	0.0236 (6)	
H14	0.190380	0.413915	0.435972	0.028*	

### Atomic displacement parameters (Å^2^)

**Table:**

	*U*^11^	*U*^22^	*U*^33^	*U*^12^	*U*^13^	*U*^23^
Br1	0.01833 (12)	0.02119 (13)	0.0434 (2)	−0.00217 (10)	0.00069 (14)	0.00037 (16)
Br2	0.02025 (12)	0.01897 (13)	0.0480 (2)	0.00223 (10)	−0.00256 (15)	−0.00061 (14)
Br3	0.02917 (16)	0.02402 (15)	0.0423 (2)	−0.00937 (12)	−0.00254 (18)	0.00189 (17)
O1	0.0460 (17)	0.0222 (13)	0.0317 (15)	0.0060 (12)	0.0009 (13)	0.0062 (11)
O2	0.0450 (16)	0.0217 (13)	0.0310 (16)	0.0042 (12)	0.0006 (13)	−0.0038 (11)
N1	0.0211 (10)	0.0185 (11)	0.0296 (15)	0.0000 (8)	0.0007 (13)	−0.0001 (12)
N2	0.0179 (10)	0.0190 (10)	0.0251 (15)	−0.0007 (8)	−0.0006 (10)	0.0012 (11)
N3	0.0184 (11)	0.0192 (11)	0.0306 (17)	−0.0012 (8)	0.0016 (10)	0.0014 (11)
C1	0.0181 (11)	0.0170 (11)	0.0315 (16)	0.0001 (9)	−0.0020 (13)	−0.0003 (13)
C2	0.0182 (11)	0.0171 (11)	0.0263 (15)	0.0002 (9)	−0.0010 (12)	0.0015 (12)
C3	0.0164 (10)	0.0167 (11)	0.0257 (15)	−0.0004 (8)	−0.0012 (13)	0.0022 (12)
C4	0.0237 (14)	0.0183 (13)	0.0243 (16)	0.0015 (11)	0.0002 (12)	0.0010 (12)
C5	0.0215 (14)	0.0211 (14)	0.0241 (16)	0.0024 (11)	0.0005 (12)	−0.0010 (12)
C6	0.0194 (11)	0.0166 (11)	0.0275 (15)	0.0000 (9)	−0.0004 (13)	−0.0007 (13)
C7	0.0262 (15)	0.0209 (14)	0.0276 (18)	0.0018 (12)	0.0018 (13)	0.0031 (12)
C8	0.0238 (14)	0.0202 (14)	0.0265 (17)	0.0003 (11)	0.0022 (13)	0.0003 (12)
C9	0.0189 (12)	0.0167 (11)	0.0269 (17)	−0.0010 (9)	−0.0007 (11)	0.0000 (12)
C10	0.0230 (14)	0.0182 (13)	0.0342 (19)	0.0003 (11)	0.0021 (13)	−0.0015 (13)
C11	0.0203 (13)	0.0209 (14)	0.037 (2)	−0.0021 (11)	0.0034 (13)	−0.0005 (13)
C12	0.0238 (13)	0.0185 (12)	0.0305 (17)	−0.0061 (10)	−0.0016 (13)	0.0016 (14)
C13	0.0246 (13)	0.0182 (12)	0.0312 (19)	−0.0007 (10)	0.0009 (14)	−0.0027 (13)
C14	0.0205 (12)	0.0208 (13)	0.0295 (19)	−0.0005 (10)	0.0035 (13)	−0.0024 (13)

### Geometric parameters (Å, º)

**Table:**

Br1—C1	1.864 (3)	C5—H5	0.9500
Br2—C1	1.868 (3)	C6—C7	1.384 (6)
Br3—C12	1.888 (3)	C7—C8	1.388 (5)
O1—N1	1.223 (5)	C7—H7	0.9500
O2—N1	1.231 (5)	C8—H8	0.9500
N1—C6	1.477 (4)	C9—C10	1.386 (5)
N2—N3	1.266 (4)	C9—C14	1.397 (5)
N2—C2	1.406 (4)	C10—C11	1.400 (5)
N3—C9	1.437 (4)	C10—H10	0.9500
C1—C2	1.352 (4)	C11—C12	1.378 (5)
C2—C3	1.492 (4)	C11—H11	0.9500
C3—C8	1.384 (5)	C12—C13	1.392 (5)
C3—C4	1.394 (5)	C13—C14	1.384 (5)
C4—C5	1.400 (5)	C13—H13	0.9500
C4—H4	0.9500	C14—H14	0.9500
C5—C6	1.379 (5)		
			
O1—N1—O2	123.8 (3)	C6—C7—H7	121.0
O1—N1—C6	118.1 (3)	C8—C7—H7	121.0
O2—N1—C6	118.1 (3)	C3—C8—C7	120.7 (3)
N3—N2—C2	115.9 (3)	C3—C8—H8	119.6
N2—N3—C9	111.8 (3)	C7—C8—H8	119.6
C2—C1—Br1	123.1 (2)	C10—C9—C14	120.6 (3)
C2—C1—Br2	122.4 (2)	C10—C9—N3	116.6 (3)
Br1—C1—Br2	114.43 (15)	C14—C9—N3	122.8 (3)
C1—C2—N2	115.0 (3)	C9—C10—C11	119.7 (3)
C1—C2—C3	122.3 (3)	C9—C10—H10	120.2
N2—C2—C3	122.7 (3)	C11—C10—H10	120.2
C8—C3—C4	120.3 (3)	C12—C11—C10	118.9 (3)
C8—C3—C2	120.5 (3)	C12—C11—H11	120.6
C4—C3—C2	119.1 (3)	C10—C11—H11	120.6
C3—C4—C5	119.7 (3)	C11—C12—C13	122.1 (3)
C3—C4—H4	120.2	C11—C12—Br3	119.2 (3)
C5—C4—H4	120.2	C13—C12—Br3	118.7 (3)
C6—C5—C4	118.3 (3)	C14—C13—C12	118.7 (3)
C6—C5—H5	120.8	C14—C13—H13	120.7
C4—C5—H5	120.8	C12—C13—H13	120.7
C5—C6—C7	122.9 (3)	C13—C14—C9	120.0 (3)
C5—C6—N1	118.3 (3)	C13—C14—H14	120.0
C7—C6—N1	118.8 (3)	C9—C14—H14	120.0
C6—C7—C8	118.0 (3)		
			
C2—N2—N3—C9	178.1 (3)	O2—N1—C6—C7	−171.8 (3)
Br1—C1—C2—N2	−178.4 (3)	C5—C6—C7—C8	−1.2 (5)
Br2—C1—C2—N2	−1.8 (6)	N1—C6—C7—C8	177.5 (3)
Br1—C1—C2—C3	1.6 (6)	C4—C3—C8—C7	0.6 (5)
Br2—C1—C2—C3	178.2 (3)	C2—C3—C8—C7	−178.5 (3)
N3—N2—C2—C1	174.8 (4)	C6—C7—C8—C3	0.6 (5)
N3—N2—C2—C3	−5.2 (5)	N2—N3—C9—C10	160.3 (4)
C1—C2—C3—C8	72.2 (5)	N2—N3—C9—C14	−20.0 (5)
N2—C2—C3—C8	−107.7 (4)	C14—C9—C10—C11	−0.9 (6)
C1—C2—C3—C4	−106.9 (5)	N3—C9—C10—C11	178.9 (4)
N2—C2—C3—C4	73.1 (5)	C9—C10—C11—C12	−0.9 (6)
C8—C3—C4—C5	−1.3 (5)	C10—C11—C12—C13	1.7 (7)
C2—C3—C4—C5	177.8 (3)	C10—C11—C12—Br3	−177.8 (3)
C3—C4—C5—C6	0.8 (5)	C11—C12—C13—C14	−0.8 (6)
C4—C5—C6—C7	0.5 (5)	Br3—C12—C13—C14	178.8 (3)
C4—C5—C6—N1	−178.2 (3)	C12—C13—C14—C9	−1.0 (6)
O1—N1—C6—C5	−172.7 (3)	C10—C9—C14—C13	1.8 (6)
O2—N1—C6—C5	6.9 (4)	N3—C9—C14—C13	−177.9 (4)
O1—N1—C6—C7	8.6 (5)		

### Hydrogen-bond geometry (Å, º)

**Table:**

*D*—H···*A*	*D*—H	H···*A*	*D*···*A*	*D*—H···*A*
C10—H10···Br1^i^	0.95	2.89	3.530 (4)	126

Symmetry code: (i) *x*−1/2, −*y*+3/2, *z*.
